# Inflammatory mediators in major depression and bipolar disorder

**DOI:** 10.1038/s41398-024-02921-z

**Published:** 2024-06-08

**Authors:** Sara Poletti, Mario Gennaro Mazza, Francesco Benedetti

**Affiliations:** Psychiatry and Clinical Psychobiology Unit, Division of Neurosciences, IRCCS San Raffaele Scientific Institute, Milan, Italy

**Keywords:** Predictive markers, Depression

## Abstract

Major depressive disorder (MDD) and bipolar disorder (BD) are highly disabling illnesses defined by different psychopathological, neuroimaging, and cognitive profiles. In the last decades, immune dysregulation has received increasing attention as a central factor in the pathophysiology of these disorders. Several aspects of immune dysregulations have been investigated, including, low-grade inflammation cytokines, chemokines, cell populations, gene expression, and markers of both peripheral and central immune activation. Understanding the distinct immune profiles characterizing the two disorders is indeed of crucial importance for differential diagnosis and the implementation of personalized treatment strategies. In this paper, we reviewed the current literature on the dysregulation of the immune response system focusing our attention on studies using inflammatory markers to discriminate between MDD and BD. High heterogeneity characterized the available literature, reflecting the heterogeneity of the disorders. Common alterations in the immune response system include high pro-inflammatory cytokines such as IL-6 and TNF-α. On the contrary, a greater involvement of chemokines and markers associated with innate immunity has been reported in BD together with dynamic changes in T cells with differentiation defects during childhood which normalize in adulthood, whereas classic mediators of immune responses such as IL-4 and IL-10 are present in MDD together with signs of immune-senescence.

## Introduction

Mood disorders have been recognized by the World Health Organization as a major source of disability, morbidity, and mortality worldwide [1]. Among mood disorders, major depressive disorder (MDD) and bipolar disorders (BD) are the most frequent and disabling ones. The lifetime prevalence is about 12% for MDD and 2% for BD [2]. Despite the partially shared clinical presentation during depressive episodes, numerous elements distinguish the two disorders and point to a different pathophysiology. BD is characterized by the presence of manic or hypomanic symptomatology, is highly heritable (approximately 70–80%), tends to have an earlier age of onset and a higher recurrence risk [3]. Lithium, the mainstay of BD treatment, is considered to be a second-line add-on drug in MDD while antidepressants, the first-line treatment of MDD, have dubious clinical efficacy in BD and might have deleterious effects [4]. Differential diagnosis between MDD and BD is usually complicated by the fact that in BD a depressive episode is often its first clinical manifestation [5, 6] and nearly 40% of BD patients are initially misdiagnosed as MDD [7], thus leading to a first-line antidepressant monotherapy that could aggravate the outcome of BD [8, 9].

**Fig. 1:**
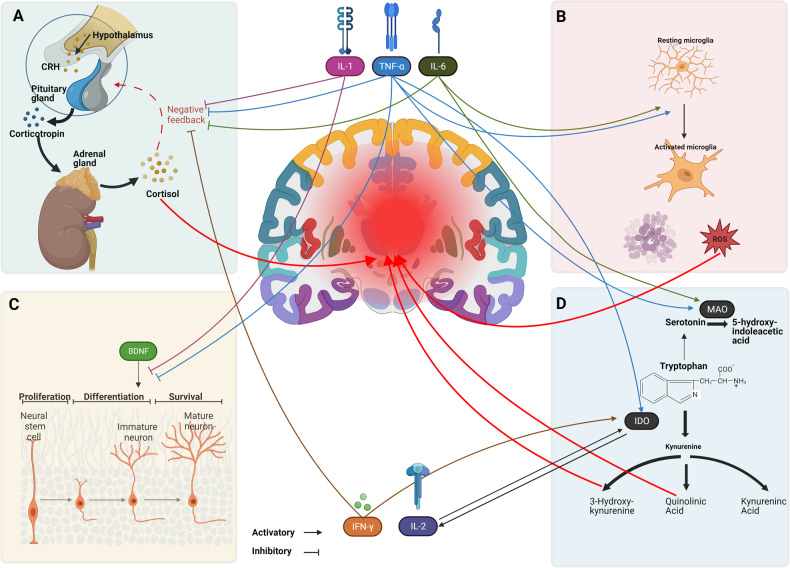
Pathophysiological mechanisms involved in mood disorders. **A** HPA axis. In case of inflammation, in response to pro-inflammatory cytokines (especially IL-1, IL-6, TNF-α, and IFN-α), there is increased secretion of corticotrophin-releasing hormone, adrenocorticotropic hormone, and cortisol [157, 158]. Normally, glucocorticoids then act as negative feedback on the inflammatory response [159] to avoid the deleterious effects of excessive production of inflammatory mediators. However, in case of prolonged inflammation (i) chronic high levels of glucocorticoids cause resistance to glucocorticoid feedback on the HPA axis, thus allowing pro-inflammatory signaling pathways to avoid normal feedback inhibition [160]; (ii) the pro-inflammatory cytokines themselves decrease the expression, translocation and downstream effects of glucocorticoid receptors, thereby blunting the negative feedback loop of the HPA axis allowing for further elevation of cortisol levels [161]. Accordingly, increased cortisol levels that are resistant to regulatory feedback by the HPA axis are among the most consistently replicated markers of mood disorder [62]. **B** Microglia. Neuroinflammation can induce microglial activation. Under physiological conditions, microglia monitors the integrity of synapses [162], removes apoptotic and necrotic cells, and promotes the maintenance of synaptic homeostasis [163]. In turn, microglial activation amplifies the innate immune response by the secretion of pro-inflammatory cytokines such as TNF-α, IL-1β, and IL-6 [164], thus increasing the production of reactive oxygen and nitroxygen species [165]. Currently, it seems that the dualistic classification of microglia activation is not fully representative of the wide repertoire of microglial states and functions in development, plasticity, aging, and disease [166], and further research is then needed to clarify the role of microglia in mood disorders. Prolonged microglial activation induces pathological neuronal apoptotic mechanisms destroying functional neuronal pathways and inhibiting the construction of new pathways, which may manifest in reduced neural plasticity and brain connectivity [167], leading to suboptimal brain function and maladaptive behaviors [168, 169]. Of notice, microglial activation in mood disorders, despite associating with depressive psychopathology, could also play a protective role in counteracting the detrimental effects of yet undefined brain insults, as observed in some brain inflammatory conditions [170]. Increased microglial activation in association with high levels of IL-6 and TNF-α has been observed in mood episodes [171]. PET studies of 17-kDa translocator protein (TSPO) binding, confirmed the presence of a microglial activation both during acute illness episodes and in euthymia [93, 172, 173]. Moreover, microglia activation was found to be associated with cognitive dysfunctions [174], the severity of depression [175], and suicide [176], in agreement with post-mortem findings of higher density of activated microglia in patients with mood disorders who died of suicide [177]. Inhibiting microglial activation with the broadly anti-inflammatory minocycline leads to better neuroplasticity and normalization of the kynurenine pathway in animal models of depression [153, 154], whereas, in treatment-resistant patients with activated peripheral markers of inflammation [155] it has an antidepressant effect. **C** Plasticity. In the case of inflammation, synaptic plasticity, learning, and memory are inhibited [178]. Pro-inflammatory cytokines affect the availability of brain-derived neurotrophic factor (BDNF), which is the main responsible for structural and functional cellular support [179]. Changes in BDNF levels have been widely reported in depression and have been suggested to underlie the behavioral and mood changes observed in the disorder [180]. Another marker of neuroplasticity is S100B whose effects depend on its concentration. Nanomolar concentrations are associated with the activation of growth and differentiation of neurons and astrocytes whereas micromolar concentrations cause apoptosis of cells, can be neurotoxic, and stimulate the expression of pro-inflammatory cytokines [181]. High serum S100B levels have been reported in major depressive and manic episodes but not in current euthymic mood disorder suggesting glial involvement in the pathogenesis of mood disorders [182]. **D** Kynurenine Pathway. Inflammatory cytokines (more specifically Interleukin (IL)-2, tumor necrosis factor (TNF)-α, and interferon (IFN)-δ) increase the conversion of tryptophan to kynurenine by activating the indolamine 2,3-dioxygenase [183, 184]. This mechanism causes the depletion of tryptophan and a subsequent decrease in serotonin levels [185]. Moreover, kynurenine degradation leads to the formation of 3- hydroxykynurenine (3-HK) and quinolinic acid (QUIN) or kynurenic acid (KA) [185]. While KA shows a neuroprotective effect, competitively antagonizing NMDA glutamate receptors, 3-HK and QUIN seem to exert neurotoxic effects [186]. Furthermore, IL-6 and TNF-α have been shown to directly increase serotonin turnover by facilitating its release and conversion into 5-hydroxyindoleacetic acid [187, 188]. Of note, serotoninergic abnormalities are a well-established feature of mood disorders pathophysiology [189, 190], with a relative decrease in cortical 5-HT stimulation during depression [191], and excessive activation of the kynurenine pathway, which seems to be shifted towards its neurotoxic branch and contribute to lower the availability of 5-HT [192, 193]. Furthermore, TNF-α, IL-8, IFN-γ, and activation of the kynurenine pathway, have been associated with white matter microstructure alteration in mood disorders [127–130], a phenotype linking response to antidepressant treatment [101, 131, 132], cognitive impairment [133], exposure to childhood traumatic experiences [134, 135], and severe depressive psychopathology [136]. Effects of inflammatory markers on white matter could also contribute to the association of brain microstructure with depression, as observed in depressive syndromes secondary to medical illnesses involving increased systemic inflammation [194].

During the last years, increasing evidence for the involvement of immune dysregulations in mood disorders has been accumulating [10–13] with a focus on the inflammatory response system (IRS), suggesting that an activation of the IRS should be considered as one of the main pathological underpinnings of mood disorders [14]. Furthermore, activation of the IRS with an overproduction of inflammation-regulating cytokines has been shown to affect different mechanisms associated with mood, emotion, and cognition, including neurotransmission, microglial activation, HPA dysregulation, and brain plasticity [15–17] (For a summary see Fig. 1).

When directly looking for specific biomarkers of activation of the IRS in mood disorders, available literature seems to converge towards a framework characterized by alterations in both innate and adaptive immunity with increased inflammatory gene expression in blood cells and higher serum levels of both pro-inflammatory and anti-inflammatory cytokines. To date, few studies tried to discriminate MDD from BD based on differences in immune responses. However, the comprehension of distinct immune profiles characteristic of these two disorders holds paramount significance for facilitating accurate differential diagnoses and the development of personalized treatment strategies.

Thus, the aim of this narrative review is to critically review the current body of literature concerning immune and IRS abnormalities focusing on the most studied biomarkers, e.g. C-reactive protein (CRP), blood cell counts, pro- and anti-inflammatory cytokines, chemokines, monocyte gene expression and T cell subset determinations. We will briefly summarize consistent findings observed separately in MDD and BD patients to provide some background to subsequentially specifically focus on studies utilizing these markers to discriminate between MDD and BD.

## Methods

We searched PubMed and ScienceDirect databases for articles up to December 2022. The search was restricted to papers written in English. We included original studies, reviews, and meta-analyses investigating the role of CRP, blood counts, cytokines and chemokines, gene expression, and immune cell populations in mood disorders, especially focusing on studies directly comparing MDD vs BD. We included only peer-reviewed articles; gray literature was excluded. Due to the multifaceted nature of the research question, the inclusion criteria were non-systematic, and papers were included from the query results or from the references cited in the selected articles. All figures were prepared using BioRender.

### C-reactive protein

CRP is a pentameric acute-phase protein whose circulating concentrations rise in response to the activation of the innate humoral system due to a wide range of acute and chronic inflammatory conditions [18]. CRP is produced by hepatocytes following IL-6 secretion by macrophages and T cells, playing a primary role in innate immunity as an early defense system [19]. CRP usually does not freely cross the BBB, however, in case of inflammation, it can pass and interact with the CNS [20, 21]. Moreover, CRP itself seems to cause BBB disruption through the binding with some ligands (i.e., Fc gamma receptors, CD16, CD32) expressed in astrocytes and endothelial cells [22]. In the context of mood disorders, different meta-analyses have investigated CRP in MDD and BD.

#### MDD

In MDD, a cumulative meta-analysis confirmed higher mean levels of CRP in patients compared to controls [23] with a moderate heterogeneity (*Q* = 51, *p* < 0.0001, I2 = 62%). In drug-naïve first-episode MDD patients [24] a meta-analysis of 5 studies comprising 179 patients and 210 HC, found that CRP in MDD drug-naive patients was higher (*g* = 0.53, 95% CI = 0.24–0.82, *P* < 0.001). Interestingly, a recent study positively correlated circulating CRP levels with cerebrospinal fluid (CSF) levels of CRP and cytokine receptors/antagonists and depressive symptom severity in patients with the highest levels [20].

#### BD

A meta-analysis investigating 23 original studies and including 1737 BD patients and 81932 HC, found that BD had higher CRP concentration [25]. When subgrouping for bipolar mood state, CRP concentrations were moderately increased in people with a depressive episode (*g* = 0.67, 95% CI = 0.23–1.11; *p* = 0.003; 11 studies, 408 patients vs 955 HC) and euthymia (*g* = 0.65, 95% CI = 0.40–0.90; *p* < 0.0001; 17 studies, 829 patients vs 79808 HC) and more substantially increased during mania (*g* = 0.87, 95% CI = 0.58–1.15; *p* < 0.0001; 14 studies, 500 patients vs 1169 HC). The extent of the increases in CRP concentrations was not related to symptom severity, while CRP concentrations were higher in patients who were not taking psychiatric medication. Finally, CRP concentration was found to decrease after the resolution of a depressive or manic episode. Another meta-analysis based on 37 studies and a total of 2215 BD patients and 3750 HC [26] reported elevated CRP levels in BD compared to HC (*g* = 0.70, 95% CI = 0.31–1.09). When considering euthymic, depressive, and manic episodes separately, CRP was elevated in both depressive and manic episodes but not in euthymia. Meta-regression showed that gender (*r* = −0.02, *p* = 0.021) and duration of illness (*r* = −0.116, *p* = 0.046) affected the CRP levels and a meta-analysis of 51 studies reported a positive association between CRP and depression (*d* = 0.22, 95% CI = 0.15, 0.28, *p* < .001) in the presence of considerable heterogeneity [27].

#### MDD versus BD

No meta-analysis has investigated CRP differences between MDD and BD, but a growing literature is now emerging. Bipolar depressed patients (*n* = 191) had an adjusted OR for inflammation (CRP > 3 mg/L) of 1.44 (95% CI = 0.6–3.4), while unipolar depressed patients (*n* = 332) had an adjusted OR for inflammation of 0.25 (95% CI = 0.1–0.6) [28]. During acute mood episodes, CRP levels in BD (*n* = 176) were significantly higher compared to MDD (*n* = 86). In the post-treatment remission phase, CRP levels were comparable among the diagnostic groups (*p* = 0.726). The predictive analysis to differentiate bipolar vs unipolar depressive episodes basing on CRP level during acute episodes achieved an AUC of 0.676 and sensitivity and specificity values of 0.636 and 0.737, respectively [29]. A predictive study found that CRP, together with age, direct bilirubin, lactic dehydrogenase, and free triiodothyronine was able to discriminate between adolescent MDD (*n* = 160) vs BD (*n* = 101) patients with a balanced accuracy of 73% and an AUC of 0.78 [30]. BD patients showed a higher rate of increased CRP than MDD (respectively 47.4% vs 36.5%) [31]. CRP levels of 6.21 mg/L have been suggested to discriminate between MDD and BD-II in both depressed and euthymic states [32].

##### CRP

These findings suggest a greater involvement of the IRS in BD as compared to MDD. However, the involvement is not stable but fluctuates together with the phases of the illnesses. Also, recent literature indicates that childhood trauma plays a role in activating the IRS [33].

### Blood counts

Blood count ratios, nominally Neutrophil/Lymphocyte ratio (NLR), Platelet/Lymphocyte ratio (PLR), and Monocyte/Lymphocyte ratio (MLR), are low-cost and reproducible markers of IRS activation [34]. Blood count ratios were found to correlate with established markers of inflammation such as CRP and IL-6 [35] and to be less affected by confounding conditions than neutrophils, platelets, or lymphocytes separately [36]. Neutrophils are critical for the first line of immune defenses regulating the innate response through phagocytosis, apoptotic action, chemotaxis, oxidative stress, and secretion of inflammatory mediators [37]. Platelets are a non-specific first-line inflammatory marker that mediate the recruitment of neutrophils and macrophages and their effector functions. Platelet activation is mediated by serotonin, dopamine, glutamate, cytokines, and P-selectin, all of which play generally an important role in psychiatric disorders [38]. Peripheral monocyte count can be considered as a sign of chronic inflammation and might be an indirect marker of microglia activation in the CNS, monocytosis seems to be linked with high levels of activated brain microglia [39, 40]. Finally, lymphocytes are primarily involved in adaptive immunity. Antibody responses are mediated by B lymphocytes whereas cell-mediated immune responses are carried out by T cells which can show a regulatory (anti-inflammatory), an inflammatory or protective function [41]. These ratios are used as biomarkers of inflammation both in medical and neuropsychiatric disorders [42–44].

#### MDD

One meta-analysis focused on MDD showing that patients had higher NLR as compared with HC (SMD = 0.670; *p* = 0.028; I2 = 89.931%) [42].

#### BD

Bipolar patients had higher NLR as compared with HC (7 studies, 693 BD vs 641 HC, SMD = 0.672; *p* < 0.001; I2 = 82.4%) and higher PLR (4 studies, 431 BD vs 368 HC, SMD = 0.425; *p* = 0.048; I2 = 86.53%). Subgroup analysis evidenced an influence of the bipolar phase on the overall estimate with studies including subjects in manic and any bipolar phase showing a significantly higher NLR and PLR as compared with HC whereas the effect was not significant among studies including only euthymic bipolar subjects. The manic phase of BD is consistently associated with increased neutrophil levels sometimes in conjunction with reduced lymphocyte levels [45]. Less consistent findings have been reported in euthymic bipolar patients who seem to have lower NLR than manic patients.

#### MDD versus BD

Single studies compared blood cell count between BD and MDD. In a recent large-scale cross-sectional study including 16,174 Chinese affective disorder patients, BD, compared with MDD patients, had higher NLR and MLR and lower PLR, although, MLR was a risk factor for both MDD and BD [46, 47]. However, bipolar patients were younger than MDD and PLR was significantly associated with age. On the contrary, a cross-sectional study using electronic health records of 13,888 psychiatric patients, did not find NLR to differ between bipolar affective disorder and depression after correcting for covariates [48].

Although few studies directly compared MDD and BD the available data are in agreement with those on the CRP in showing a greater activation of the IRS in BD compared to MDD. The altered PLR in BD suggests a greater endothelial dysfunction which could affect the trafficking of peripheral inflammatory markers to the brain [49].

##### Blood counts

The findings derived from meta-analyses and single studies comparing blood count ratios also consistently confirm that mood disorder patients present with an activation of the IRS when compared with HC. Moreover, a growing literature suggests a stronger activation in BD patients than in MDD patients and, interestingly, an even stronger activation in BD manic patients than in BD-depressed patients.

The stronger abnormalities of the PLR in BD as compared to MDD suggest a greater involvement of endothelial stickiness and permeability in this disorder.

### Cytokines and chemokines

Cytokines are small free and membrane-bound proteins that act as signaling molecules to regulate immune cellular activities and inflammation [50]. Cytokines are produced by both immune (e.g., macrophages, lymphocytes, mast cells) and non-immune (e.g., parenchymal cells, endothelial and epithelial cells, fibroblasts, adipocytes, and stromal cells) cells and the same cytokine can be produced by multiple cell types [51]. In the brain, cytokines are produced by microglia, astrocytes, endothelial cells, and neurons [52]. Further, cytokines from the periphery can affect inflammatory processes in the brain via several pathways including humoral (passing the BBB), neural (through the vagus nerve stimulation), and cellular (through the recruitment of leukocytes to the brain) [53]. Physiologically, cytokines are involved in brain development and support neuronal integrity, neurogenesis, and synaptic remodeling [54]. Cytokines can also promote behavioral alterations by influencing neurocircuitry and neurotransmitter systems [55].

Chemokines are a group of secreted proteins within the cytokine family involved in cell migration. By stimulating the migration of cells, they are crucial for immune surveillance as well as for inflammatory reactions. Their function can be either homeostatic, inflammatory, or with a dual function. Homeostatic chemokines are expressed at constitutive levels and are required for basal immune cell migration at a steady state, while inflammatory chemokines are expressed under the control of pro-inflammatory factors, attract immune cells to the site of inflammation [56, 57], induce the release of pro-inflammatory mediators, and aid in the control of T helper (Th)1/Th2/Th17 phenotypic polarization [58].

As indicated above, changes in the activation of the IRS parallel mood fluctuations in acute illness episodes. These episodes of IRS activation show increased levels of both inflammatory and anti-inflammatory cytokines. Antidepressant treatments may then enhance the activity of anti-inflammatory cytokines promoting the return to euthymia. However, immune alterations have also been reported in euthymic patients, suggesting a persistent activation of the IRS with some cytokine alterations becoming apparent during symptomatic relapses while others persist independently of the phase of the illness.

Although several studies have been performed investigating immune/inflammatory markers in depression and across mood states, it is difficult to draw a coherent picture because of contrasting findings in a plethora of tested cytokines/chemokines and their soluble receptors (see Table 1). The majority of the studies performed to investigate these markers in unipolar and bipolar depression focused only on a small number of specific cytokines/chemokines, and there is a great variety in the studies. Conflicting results have been obtained.

**Table 1:** Direction of differences in cytokines and chemokines between MDD and BD also according to mood episodes.

	MDD vs HC	BD vs HC	Euthymia	Depression vs euthymia	Depression vs mania	Mania vs euthymia
CRP	↑	↑	↑			
IL-6	↑	↑	↑		↓/↑	↑
sIL-6	-	↑				
TNF-α	↑	↑	↑-	-	-	
sTNFR2	↑					
sTNRF1		↑	↑-	↓	↓	
sTNFR80					↓	↑
IL-4	-	↑ (Mania)	↑		↓	
IL-10	↑					
CCL2	↑					
CCL3	-					
CXCL8 (IL-8)	-/↑	↓	↑ (CSF)			
CXCL10		↑	↑			
CCL11		↑ (Mania)	↑			

In this review, we will focus on just a few cytokines and chemokines, i.e. IL-6, TNF, IL-10, IL-4, CCL2, and IL-8, on which there is consistent literature. We will thereafter review the few studies that directly compared MDD versus BD in studies with larger panels of cytokines and chemokines.

### The inflammatory cytokines IL-6 and TNF-α

#### MDD

A recent meta-analysis of 82 studies performed in 6010 participants (3212 participants with MDD and 2798 HCs) reported consistent increased levels of the pro-inflammatory cytokines IL-6 (*g* = 0.621; *P* < 0.001) and TNF-α (*g* = 0.638; *P* < 0.001) in MDD compared to HC [59]. Interestingly, different biomarker profiles could be associated with subtypes of MDD, with elevated levels of TNF in atypical depression (characterized, according to DSM-5, by a higher reactivity to environmental stressors) [60], and mixed findings in melancholic depression [59], less reactive to the environment, and with a higher HPA axis activation which might influence peripheral levels of inflammatory markers [61–63]. Interestingly, elevated levels of plasma TNF-α were found in patients with atypical depression [60], but not in patients with melancholic depression [59]. This finding is in line with the hypothesis that melancholic depression is associated with increased activity of the HPA axis which would lower inflammation [61, 62].

#### BD

Studies directly comparing different mood states in BD showed similarly high TNF-α levels [64, 65]. Regarding IL-6, higher levels have been found in manic/hypomanic rather than in depressive and euthymic patients. However, the literature is not clear on relationships with BD phases [66], because also, both a significant decrease in IL-6 [64] levels in manic compared with depressed patients and negative findings of a difference between mood states have been reported [65, 67].

Different studies focused also on TNF receptors showing higher levels of sTNFR1 in both active phases and the euthymic state compared to the depressive state [68, 69]. Also, higher levels of sTNFR80 [70] have been found in manic/hypomanic rather than in depressive and euthymic patients. Finally, an association between these markers and both depressive and manic symptoms has been reported [66]. All in all, recent independent meta-analyses suggest a shared inflammatory profile between both depressive and manic stages characterized by high concentrations of IL-6 and TNF-α [26, 66, 71, 72].

Both higher and lower markers have been reported in euthymic patients. Findings from systematic reviews and meta-analyses showed a permanent increase of IL-6 [73], and unaltered levels of TNF-α [73, 74], whereas contrasting findings have been reported on sTNFR1 [72–74].

### The anti-inflammatory/regulatory cytokines IL-10 and IL-4

#### MDD

Higher levels of IL-10 were consistently reported in MDD compared to HC [59]. Furthermore, IL-10 levels were not significantly altered in patients who were antidepressant-free but only in participants who were using antidepressants. This seems to suggest that an antidepressant regimen would boost IL-10-mediated immunosuppression and reduce inflammation.

#### BD

Increased levels of anti-inflammatory cytokines other than IL-4 are scarcely reported in BD. Regarding IL-4, the consistent finding is an increase in IL-4 during manic phases [71, 72, 75].

Studies directly comparing different mood states showed a significant increase in IL-4 levels [64] in manic compared with depressed state patients. And in euthymic compared to HC [76].

Finally, negative findings of a difference between mood states have been reported for IL-10 which resulted in higher compared to controls in both groups of patients [67].

### The chemokines CCL2 and IL-8

#### MDD

There is also evidence that chemokine alterations occur in MDD. Increased peripheral levels of CCL2 have been observed in MDD [59].

Similarly, increased peripheral [77, 78] and central [79] levels of IL-8 have been reported although less consistently possibly due to age and medication effects as suggested by higher levels in females but not in males [80]. A meta-analysis also showed that peripheral IL-8 levels were significantly decreased in drug-naïve first-episode MDD patients compared with controls [24].

#### BD

The most consistent findings in BD are: increased CCL2 [68, 81] and IL-8 [82] levels in depressed BD compared to HC [83]; and lower [68] levels of IL-8 in manic patients compared to controls. Further, although no association was observed with values measured in the peripheral blood of euthymic patients, increased IL-8 levels have been reported in the CSF [84].

### MDD versus BD

Few studies, implementing both conventional and machine learning methodologies, considered cytokines and chemokines as possible tools to differentiate BD from MDD. Using elastic net penalized logistic regression, we recently showed that BD was predicted by the levels of several cytokines and chemokines including TNF-α, CCL3, CCL4, CCL5, and CCL11; while MDD was predicted mainly by the levels of IL-4, IL-6, and IL-10 with a sensitivity of 79% and a specificity of 99% [83].

With a similar approach and with a sensitivity of 0.77% and specificity of 0.76% IL-6 and IL-10 were associated with BD in a discovery cohort, whereas in a replication cohort, IL-10 was confirmed and TNF-α, CCL3, and CCL11 individuated [85].

Also, higher IL-6, sTNFR2, and lower TNF-α have been identified as predictors of BD through a hierarchical logistic regression, reaching 98.1% accuracy [86].

Considering 10 initial analytes and integrated feature selection, performed through a multivariate wrapper technique, with a support vector machine with a linear kernel and leave-one-subject out cross-validation IL-4 and IL-10 were able to discriminate BD from MDD with a sensitivity of 62% and specificity of 66% [87].

#### Cytokines and chemokines

A great heterogeneity characterizes the study of cytokines and chemokines in mood disorder patients reflecting the technical heterogeneity of the used assays, the intrinsic heterogeneity of the two disorders with influences of body weight and childhood trauma on the levels of the cytokines, the episodic nature of these illnesses, and the variable effects of drug treatment. So, conclusions are very difficult to draw from the cytokine/chemokine studies, regarding differences between MDD and BD.

Both MDD and BD are characterized by increased pro- and anti-inflammatory cytokines. The studies directly comparing BD versus MDD show higher levels of certain chemokines (CCL3 and CCL11, in two studies) in BD compared to MDD; also, the chemokine IL-8 is prominent in BD. This suggests a greater involvement in the trafficking of cells in BD.

### Monocyte gene expression

Monocytes/macrophages are important producers of pro-and anti-inflammatory cytokines and chemokines. The expression of genes regulating inflammatory processes has been extensively studied in monocytes of BD and MDD patients [88]. These studies were not limited to a rostrum of strictly inflammatory genes (such as IL-1, IL-6, CCL2, CCl20, TNF, etc.), but also extended to genes playing a role in the differentiation, apoptosis, mitochondrial function, and lipid metabolism of the cells (such as BAX, BCL10, EGR1, EGR2, NR1H3, ABCA1, ABCG1, MVK, and HMOX). All these genes had been found in whole genome analysis studies discriminating monocytes of MDD and BD patients from those of healthy controls.

#### MDD

In MDD patients monocytes show an abnormal expression of genes involved in pathways implicated in apoptosis, growth, lipid metabolism, and redox reactions, suggesting a mitochondrial dysfunction and an early aging of the cells [14].

However, in cases of a history of childhood trauma and/or in cases above 30 years of age, there is an additional overexpression of a variety of strictly inflammatory genes involved in the regulation of the IRS via the expression of a number of excitatory and inhibitory genes, amongst which e.g. IL-6, IL-1, CCL20, CXCL2 and DUSP2 and TNFAIP3 [14].

In younger mild MDD cases of less than 30 years with a first episode of depression without signs of anxiety, even a reduced expression of these strictly inflammatory genes was found [89].

#### BD

In the manic and depressive phases of BD, an increased number of circulating monocytes has been reported [90]. These monocytes show overexpression of a variety of the strictly inflammatory genes involved in the regulation of the IRS via the expression of the same excitatory and inhibitory genes as in MDD.

In euthymic phases of the disorder the overexpression of the inflammatory genes is absent in monocytes [91], and in fact in one report there even is a decrease in the expression of such genes as compared to healthy controls [92].

Also, in mania and bipolar depression PET studies [93] focusing on the activation of microglia report an activated status with an increased PET signal in several regions including the cingulate cortex and the hippocampus.

#### MDD vs BD

Although no study directly compared monocyte gene expression between MDD and BD, a study investigated the expression of TREM-1 and PU.1 in monocytes of melancholic MDD patients and BD patients with active disease compared to controls [94]. TREM-1 is an important membrane-bound molecule involved in inflammation regulation [95], while PU.1 is a transcription factor important for monocyte/macrophage development and differentiation [96].

TREM-1 gene expression was significantly increased in monocytes of BD patients, while in MDD patients TREM-1 expression only tended to be overexpressed. In contrast, PU.1 gene expression was increased in the monocytes of MDD patients, while it was not increased in the monocytes of BD patients.

##### Monocyte gene expression

Pieces of evidence show the presence of monocyte activation both in the periphery and the brain in both disorders. Monocytes gene expression suggest that in BD monocytes are more involved in inflammation regulation than the monocytes of MDD patients, while the monocytes of MDD patients are more involved in the regulation of growth and differentiation.

### T cell populations

Extensive studies have been performed on subpopulations of T helper cell subsets involved in regulating the inflammatory response in mood disorders (Table 2). T cells are actors of adaptive immunity and together with NK cells contribute to and regulate the cell-mediated immune response. However, the role of monocytes/macrophages and T helper cells within the CNS are not limited to immune and inflammatory response regulation, but also includes the regulation of neurogenesis [97]; further they have been shown to play a role in the maintenance of cognition [98], learning and behavior [99]. Adaptive and innate immune support, ensured by cell trafficking across the BBB, is essential for brain maintenance and repair in healthy conditions, and it is disrupted in several brain disorders [100]. There is a crosstalk between neurons and leukocytes, influencing brain function and structure in mood disorders [101]. Although the field is in its infancy, the few available studies in mood disorders suggest that immune cells influence brain white matter microstructure [102–104], and are in turn influenced by pharmacological treatments also partially mediating their effects on the brain [103], thus supporting the study of circulating immune cell composition and activation status as a new promising biomarker. The post-mortem observed abnormal density of T lymphocytes in subgroups of mood disorder patients is consistent with this perspective [105]. Both post-mortem and brain imaging studies also suggest an activation of microglia in patients with mood disorders [39]. Finally, premature aging was observed both in BD (increased proportion of late‐differentiated T cells and reduced proportion of activated T cells after stimulation) which is associated with the number of episodes [106], and MDD, especially in those patients exposed to childhood adversity who show higher signs of inflammation [14], with the expansion of senescent memory T cells [107].

**Table 2:** Direction of differences in gene expression and T cell populations between MDD and BD also according to mood episodes.

	MDD vs HC	MDD with CT or older age	BD vs HC	Euthymia
Monocytes IRS genes	-	↑↑	↑↑	-/↓
Monocytes CIRS genes	-	↑↑	↑↑	-/↓
Monocytes metabolic/growth genes	↑	↑		
T cells	-	-		↑
Th2	-	↓	-	↑
Th17	-	↓	-	↑
Tregs	-	↓	↑ (Young)	↑/-

*CT* childhood trauma.

#### MDD

##### Th1/Th2

An increase in the Th1/Th2 ratio in the peripheral blood of MDD patients was reported [108], together with a decrease in Th2 cells [91]. However, a recent meta-analysis reported reduced percentages of both Th1 and Th2 [109]. In remission phases, the number of T cells seems to remain unchanged but the in vitro proliferative status of these cells is diminished. Further, T cells undergo apoptosis to prevent uncontrolled proliferation suggesting that T cells may adapt to MDD [110].

#### Th17 cells

High levels of Th17 cells have been reported in severe forms of MDD, characterized by high depression scores and high suicide risk [111]. However, in less severe forms Th17 counts are normal, and generally, MDD patients are characterized by impaired maturation of Th17 cells (and of Th2 cells and NK cells) and by decreased serum levels of the T cell differentiation factor IL-7 and the T cell activation marker sCD25 [112].

#### T Regulatory cells

Reports of reduced levels of regulatory cells involve different populations including natural T regulatory cells (Treg) [108, 113, 114] and NK regs [115]. These deficiencies are paralleled by an increased inflammatory state of monocytes as a sign of chronic low-grade inflammation [107]. Antidepressant therapy increased the number of T regulatory cells [107].

#### Cytotoxic cells

A recent meta-analysis showed no differences between patients and controls for CD8 cells. Further, a higher NK absolute cell count was reported but no difference was observed for relative percentages [109].

#### BD

##### TH1/Th2

Although discrepant findings have been reported on Th1 [116] and Th2 [92] cell populations, with both higher levels of Th1 and Th2 and no difference [90] compared to controls, a recent meta-analysis suggest that an imbalance between Th1 and Th2 is present in BD with a shift towards Th2 [117].

#### TH17

Higher levels of activated CD3+ T cells have been reported more than a decade ago in both symptomatic and euthymic BD patients compared to healthy controls [118], these increases included higher levels of Th17 cells [119]. Patients with BD in a euthymic state indeed have increased levels of Th17 cells compared to healthy individuals [91, 92]. However, there is also a report not showing such increased levels of Th17 cells in euthymic BD patients [90].

To support a view of a high involvement of Th17 in BD a cytokine study has shown higher levels of IL-17 in the circulation of patients with BD in remission [120].

It is also of interest to mention that studies in offspring of a bipolar parent show that in teenage time and adulthood, Th17 cell levels follow a dynamic course as compared to Th17 levels in healthy controls with initial reduced levels in young adulthood. This suggests proneness of the T cell system of BD patients to influences of sex hormones fluctuating in teenage/young adulthood time [113].

#### T regulator cells

A very recent study shows that euthymic patients with BD show higher levels of Tregs compared to healthy controls. The literature on BD on this cell population is contradictory. An early study showed higher circulating Tregs in younger-than-40-year-old patients with BD in an active disease state [90], but decreased levels in relatively old euthymic BD patients (median 52 years, [92]). Other studies have also shown lower levels of Tregs in patients with euthymic BD [116, 121, 122].

Similar to Th17 cells dynamic changes have been observed in children of a bipolar parent who are at risk for bipolar disorder [113]. Particularly in teenage time reduced numbers of T regulatory cells were found. These reduced numbers of Tregs were associated with a high expression of pro-inflammatory genes in monocytes suggesting a high inflammatory state in teenagers with a bipolar parent; things changed in young adulthood when T cells producing pro-inflammatory cytokines and expression of pro-inflammatory genes in monocytes are reduced suggesting a transition to an anti-inflammatory state [112, 113].

#### Cytotoxic cells

A reduction of CD8 cells has been reported in BD [121] whereas no differences between BD and HC have been reported for NK cells [103].

#### MDD vs BD

Becking et al. directly compared the Th17 and Treg cell populations (as well as Th1 and Th2 populations) between MDD patients and BD patients of whom the vast majority was in euthymia. Compared to MDD patients, BD patients showed significantly increased levels of Th17, Th2, Th1, and T regulatory cells (all *p* < 0.02). Also, In BD patients, levels of Th17 and T regulatory cells were increased compared to HC (*p* = 0.03, *p* = 0.02, respectively), while MDD patients showed decreased levels of Th17 and Th2 compared to HC (*p* = 0.03, *p* = 0.01, respectively). Of the various medications only SSRI/SNRI usage could explain part of the Th2 decrease in MDD [119]. A study performed on patients during a depressive episode showed that BD patients had lower CD8+ T cell compared to MDD, while there was no difference in the CD4+ T cell [123]. Finally, Li et al. reported lower CD4+ T cell and NK cells but higher CD8+ T cell proportions in BD-depressed patients compared to MDD and a higher CD4/CD8 ratio (a marker of immune-senescence) in MDD compared to BD [124].

Thus collectively, results although not consistently suggest the presence of differences in T cell immune regulation between patients with BD and MDD.

##### Immune cell populations

A partial T cell defect starting early in adolescence, involving a reduction of naïve T cells and Treg cells and an expansion of memory and senescent T cells with a dynamic pattern of premature immunosenescence seems to characterize MDD patients, especially with childhood trauma [14, 102, 119, 125, 126]. More complicated is the picture in BD where substantial changes in the frequencies of cell populations can be observed throughout the lifespan and possibly also in different mood episodes. Differences between MDD and BD in this pattern are present according to age and phase of the illness and involve different cell populations including Th17 and Th2 which are able to differentiate the two disorders.

## Discussion

**Fig. 2:**
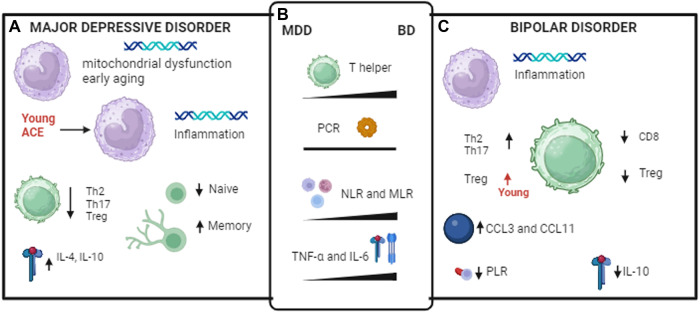
Differences in immune responses in MDD and BD patients. **A** immune/inflammatory markers associated only with MDD. **B** immune markers associated with both disorders but showing different peripheral levels. The differences between the two disorders are represented by a triangle with its vertex indicating the lower plasma level. **C** immune/inflammatory markers associated only with BD.

Although the literature agrees on the presence of distinct profiles of immune biomarkers, the characterization of these profiles is less clear (Fig. 2). Focusing only on the most consistent findings, some pro-inflammatory cytokines such as IL-6 and TNF-α have been associated with both disorders in different studies. Directly comparing the two disorders MDD has been associated with increased levels of IL-4 and IL-10, whereas BD has been associated with NLR, CRP, and several chemokines including CCL3, CCL4, CCL5, and CCL11 and, on the whole, a greater involvement of the IRS.

It’s not by chance that IL-6 and TNF-α are involved in the main psychopathological mechanism associated with mood disorder (Fig. 1). IL-6 and TNF-α have been associated with white matter microstructure alteration in mood disorders [127–130], a phenotype linking response to antidepressant treatment [101, 131, 132], cognitive impairment [133], exposure to childhood trauma [134, 135], and severe depressive psychopathology [136].

Focusing on the differences between MDD and BD we observed that the markers increased in MDD, including gene expression of monocytes, are involved in macrophage activation and Th2, Treg differentiation. In BD, increased markers are involved in monocyte/macrophage responses and cellular activation, proliferation, and migration, especially of the Th2 pathway. These patterns could also explain the reduced gray matter volumes reported in BD compared to MDD and the greater number of episodes characteristics of these patients [137].

Whereas the majority of the cytokines are involved in a monocyte/macrophage response and in the recruitment of cells responsible for the adaptive immune response, cytokines, such as IL-4 and IL-10, may protect from an over-reactive immune system, promote repair by immune-regulatory mechanisms and induce a tolerance counteracting the pro-inflammatory system. These actions may be promoted also by IL-6 which exhibits context-dependent immune-regulatory activities [138]. In line with the finding of the role of IL-4 and IL-10 in differentiating BD from MDD, Th2 cell populations have been shown to play the same role, confirming the involvement of these cell populations in mood disorders. Higher levels of Th17 also seem to associate with BD diagnosis where, although counterintuitively, correlated with higher white matter integrity, this finding, together with a lack of association of BD with IL-17 levels may be explained by the high plasticity of Th17 cells which, when induced by IL-6/TGF-b are less inflammatory and have a higher expression of IL-10 [139].

The heterogeneity observed in the literature well reflects the one characteristic of these disorders. Several factors may determine the activation status of the IRS and the mediators involved: (1) the episodic nature of these disorders with different polarities and periods of well-being; (2) the high presence of childhood trauma consistently associated with inflammation; (3) the comorbidities with metabolic disorders such as obesity, type 2 diabetes, and metabolic syndrome which also are linked to inflammation; (4) pharmacologic treatments which on the contrary may reduce inflammation. All these factors make it rather difficult to draw a coherent picture.

From a clinical point of view, in physiological conditions, cytokines interact with 5-HT to shape sleep architecture [140], and perturbing microglial functions, disrupt sleep and the homeostatic processes associated with synaptic potentiation and dendritic spine density [141, 142]. Cytokines’ release follows a circadian pattern which is dysregulated in mood disorders [143]; winter depression associated with higher macrophage activity and lower lymphocyte proliferation [144]; and antidepressant treatments targeting the biological clock normalize both immune functions and depressive symptoms [144, 145]. Disruption of rhythms across the lifespan leads to the onset, recurrence, and worsening of mood disorders [146]: it can be surmised that an abnormal immune system activation could play a key role in this process [147]. Again, the abnormalities in circadian rhythms differ in MDD and BD [148] suggesting different underlying mechanisms.

Inflammatory status has been also associated with response to antidepressant treatments in MDD [149, 150] and BD [17]. Higher levels of circulating cytokines, especially IL-6 and TNF-α, hamper antidepressant response and contribute to treatment resistance [150, 151]. Immune/inflammatory mechanisms have therefore been proposed as possible targets for antidepressant psychopharmacology, and randomized, placebo-controlled trials confirmed the potential efficacy of anti-inflammatory and immune-modulatory agents in treating depression only in subgroups of patients with increased peripheral inflammation [149]; on the other hand, conventional antidepressants share immune-modulatory and anti-inflammatory properties, which could reduce inflammation during depression [152]. Also, inhibiting microglial activation with the broadly anti-inflammatory minocycline leads to better neuroplasticity and normalization of the kynurenine pathway in animal models of depression [153, 154], whereas, in treatment-resistant patients with CRP > 3 [155] it has an antidepressant effect. In agreement with a different immune profile in MDD and BD, a recent study from our group showed how MDD and BD patients respond differently to an immune-modulatory treatment with low-dose IL-2: higher effect of treatment in BD; increase of CD4^+^Naïve T cells and decrease in CD4^+^Central Memory cells only in MDD [156]. These effects are in agreement with the different involvement of the IRS in the two disorders and support the usefulness of this perspective in clinical practice.

As already noticed, the available literature is characterized by high heterogeneity indeed, some clinical features, known to affect IRS, should be taken into account when trying to use immune markers to differentiate MDD from BD: (i) age; (ii) BD type I or II; (iii) number of previous episodes; (iv) duration of the illness; (v) pharmacologic treatment; (vi) smoking and BMI; (vii) childhood trauma.

## Conclusion

Altogether, available findings about the role of immune-related biomarkers suggest that they can provide new targets to differentiate patients subgroups among the wide heterogeneity of mood disorders, including in providing new endophenotypes for the early differentiation between bipolar and unipolar illnesses; and to address new treatments aimed at preventing the detrimental effect of the illness on brain structure and function, which appears to be related to the repeated insult caused by neuroinflammatory mechanism during the lifetime recurrence of mood episodes. Following this perspective, the differential activation of innate immunity and monocyte/macrophagic activity associated with BD could contribute to explain the worse brain integrity and cognitive deterioration observed in the illness.

The interest for further clinical and preclinical investigations is then warranted to elucidate the immune/inflammatory mechanisms as a possible target for antidepressant psychopharmacology, in order to translate the acquired knowledge into clinical practice aiming at personalized treatment regimens for depressed patients, and to further understand the most promising perspective in elucidating the pathophysiology of mood disorders.
